# A novel homozygous *DLGAP5* splice-site variant in a woman with oocyte maturation arrest and recurrent IVF failure: a case report

**DOI:** 10.3389/fgene.2026.1963011

**Published:** 2026-09-24

**Authors:** Prince Jacob, Deepa Bhat, Adithya G. Urs, Uma Maheshwari, Siddaramappa Jagadish Patil

**Affiliations:** 1 Department of Medical Genetics, JSS Medical College, JSS Academy of Higher Education Research, Mysuru, Karnataka, India; 2 Department of Genetics, Yale School of Medicine, New Haven, CT, United States; 3 Public Health Research Institute of India (PHRII), Mysuru, Karnataka, India; 4 Department of Anatomy, JSS Medical College, JSS Academy of Higher Education and Research, Mysuru, Karnataka, India; 5 Consultant Fertility Specialist, Fertility Centre - Cloud Nine Hospital, Jayanagar Bengaluru, India; 6 Division of Medical Genetics, Narayana Hrudayalaya Hospitals / Mazumdar Shaw Medical Centre, Bangalore, India

**Keywords:** case report, DLGAP5, expanding the mutational spectrum, oocyte maturation arrest, recurrent early embryonic arrest

## Abstract

Oocyte maturation arrest (OMA) is a rare cause of female infertility characterized by impaired oocyte maturation and recurrent failure of assisted reproductive technologies. Recent studies have identified *DLGAP5* as a critical regulator of meiotic spindle assembly, with biallelic variants causing oocyte maturation defects and early embryonic arrest. Till date, 10 females from seven families have been reported. We report a 27-year-old Asian - Indian woman from a consanguineous family with 5 years of primary infertility and two IVF cycles characterized by poor metaphase II (MII) oocyte yield and complete blastocyst arrest. Whole-exome sequencing identified a novel canonical splice-acceptor variant, c.2064-1G>A, in homozygous state in *DLGAP5*. Segregation analysis confirmed heterozygous carrier status in both parents, whereas the variant was absent from the apparently healthy male sibling. This report expands the mutational spectrum of *DLGAP5* and highlights the value of genomic testing in women with recurrent IVF failure and an OMA-like embryology phenotype.

## Introduction

Oocyte maturation arrest (OMA) is an uncommon cause of female infertility characterized by failure of oocytes to complete meiotic maturation, resulting in reduced metaphase II (MII) oocyte yield, and impaired embryonic development leading to recurrent failure of assisted reproductive technologies (ART). It is typically suspected in women with repeated IVF cycles demonstrating low MII yield, poor embryo cleavage, and failure to achieve blastocyst-stage embryos in the absence of other identifiable causes (Solovova and Chernykh, 2022; Zhu et al., 2022; McCoy et al., 2023).

Advances in and clinical use of next-generation sequencing have established that monogenic defects are an important cause of recurrent ART failure, particularly in patients with severe and reproducible embryology phenotypes (Chen et al., 2026). Among the recently identified genes, *DLGAP5* has emerged as a critical regulator of meiotic spindle assembly and chromosome segregation during oocyte maturation (Wang et al., 2026). Human and functional studies have shown that biallelic variants in *DLGAP5* disrupt spindle organization, impair oocyte maturation, and cause early embryonic arrest, establishing the gene as a cause of autosomal recessive female infertility (Qu et al., 2025; Hu et al., 2025; Wang et al., 2025).

Here, we report a woman from a consanguineous Asian Indian family with recurrent IVF failure characterized by poor oocyte maturation and complete blastocyst arrest, in whom whole-exome sequencing identified a novel canonical splice-site variant in homozygous state in *DLGAP5*. This case expands the mutational spectrum of *DLGAP5*, and provides additional evidence of *DLGAP5*-related female infertility from Southern India.

## Case report

A 27-year-old woman, the offspring of a consanguineous marriage, presented with primary infertility of 5 years’ duration (Figure 1A). Baseline evaluation suggested reduced ovarian reserve (AMH 0.6 ng/mL), with mildly elevated prolactin (31.8 ng/mL) and normal thyroid function (TSH 2.75 mIU/L). Pelvic ultrasonography showed a structurally normal uterus and morphologically normal ovaries with a reduced antral follicle count, and peripheral blood karyotyping demonstrated a normal female karyotype (46,XX). Her spouse was 32 years old and had teratozoospermia, with 2%–3% normal forms and a DNA fragmentation index of 12%, with a normal male karyotype (46,XY).

**FIGURE 1:**
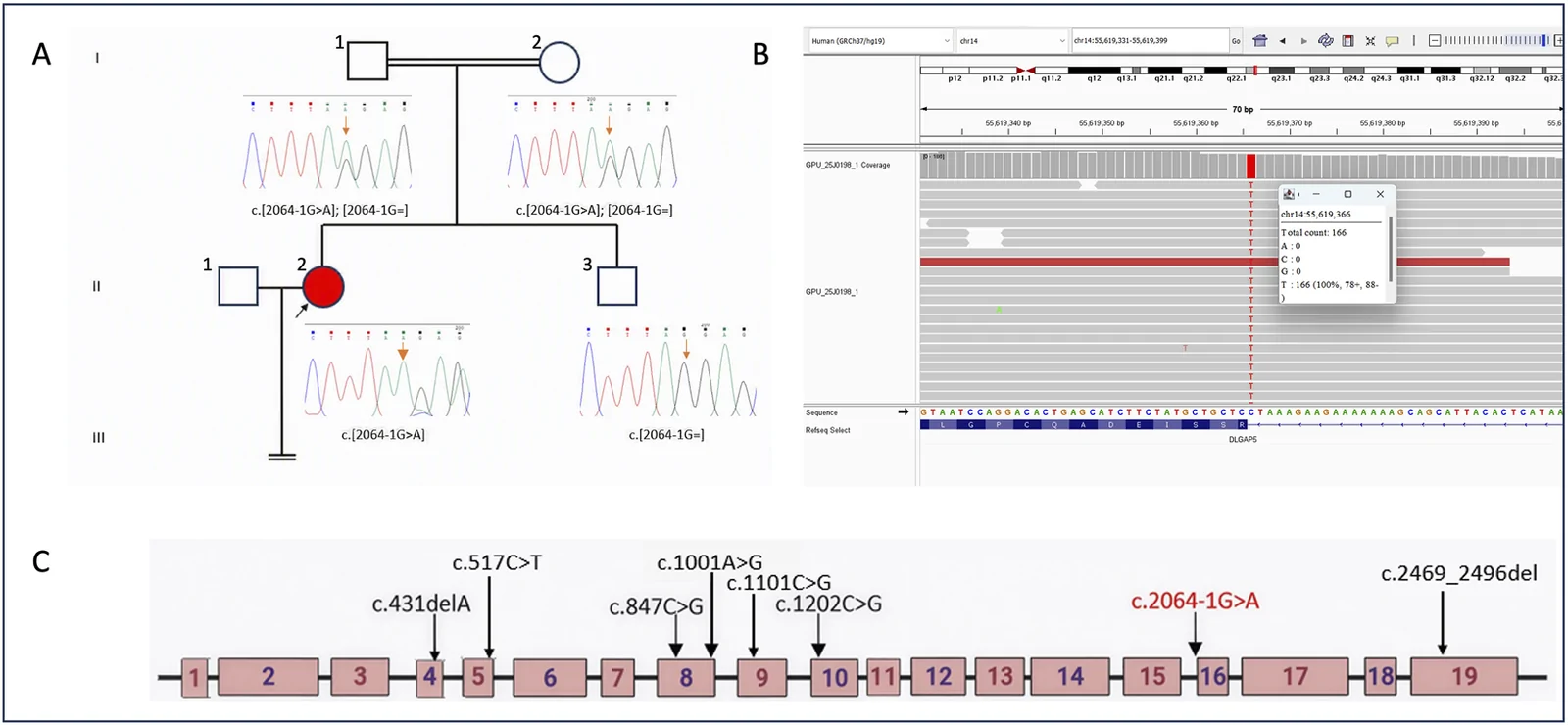
Pedigree of family **(A)** with validation and segregation results of variant *DLGAP5* (NM_014750.5)*:* c.2064-1G>A. The variant is observed in homozygous state in the proband (II.2), heterozygous state in parents (I.1 and I.2), and not observed in sibling (II.3). IGV tool screenshot **(B)** depicting reads with homozygous canonical splice variant in *DLGAP5.* Schematic representation **(C)** of *DLGAP5* (NM_014750.5) exons showing loci of previously reported disease variants and variant observed (red) in the present study.

In view of the prolonged infertility and the couple’s overall clinical profile, assisted reproductive technology was undertaken. ICSI was performed in both treatment cycles using fresh semen samples obtained independently from the same male partner for each cycle. Semen parameters and detailed stimulation and embryological data are provided in Supplementary Table 1.

Across the two stimulated treatment cycles, 11 oocytes were retrieved, of which six reached metaphase II (MII) maturity (54.5%). In the first cycle, six oocytes were retrieved, of which three were MII oocytes. Following ICSI, all three MII oocytes fertilized. Day-3 assessment showed three cleavage-stage embryos (7-cell Grade B, 5-cell Grade B, and 2-cell Grade C); however, none progressed to the blastocyst stage despite culture until Day 6. In the second cycle, five oocytes were retrieved, of which three were MII oocytes. One oocyte showed a large polar body. Two oocytes fertilized following ICSI; however, both embryos demonstrated markedly delayed cleavage, remaining at the 2-cell stage on Day 3 (Grade C), and neither progressed to the blastocyst stage despite culture until Day 6.

Overall, five of the six MII oocytes fertilized following ICSI (83.3%); however, all resulting embryos demonstrated impaired or delayed development, with complete failure of blastocyst formation across both cycles. Abnormal oocyte morphology was also documented during treatment. While diminished ovarian reserve may have contributed to the reduced oocyte yield, it did not readily explain the reproducible pattern of reduced oocyte maturation, abnormal oocyte morphology, delayed embryonic cleavage, and uniform pre-blastocyst developmental arrest. This phenotype prompted further evaluation for an underlying monogenic etiology.

During follow-up, the patient conceived spontaneously. However, serial β-hCG measurements showed a declining trajectory, and the pregnancy resolved prior to sonographic confirmation of an intrauterine gestation, consistent with a biochemical pregnancy. This outcome was recorded as part of the subsequent reproductive history; however, in the absence of genetic or embryonic evaluation, its relationship to the *DLGAP5* variant or the previously observed ART phenotype could not be established.

Genomic DNA was extracted from peripheral blood of the proband and available family members. Couple Whole-exome sequencing (WES) was performed using the Twist Human Core Exome 2.0 kit (Twist Bioscience, South San Francisco, CA, USA) for target enrichment, according to the manufacturer’s instructions. Libraries were sequenced on an Illumina NovaSeq 6000 platform using paired-end sequencing.

Raw sequencing reads were subjected to quality control and aligned to the GRCh37/hg19 human reference genome using the Burrows–Wheeler Aligner (BWA). Single-nucleotide variants and small insertions/deletions were identified using the Genome Analysis Toolkit (GATK) variant-calling pipeline. Variants were subsequently annotated and prioritized based on their predicted molecular consequence, population frequency, inheritance pattern, and phenotypic relevance.

Rare variants were filtered against population databases, including the Genome Aggregation Database (gnomAD v4.1), and variants with allele frequencies incompatible with a rare autosomal recessive disorder were excluded. Candidate variants were prioritized based on their consistency with the patient’s phenotype and the known or emerging biological role of the corresponding gene in reproductive function. Appropriate prediction tools (SIFT, Revel, Mutation Taster, Splice AI, Human Splice Finder) were assessed for each of the variants and were correlated with the patient’s phenotype.

WES identified a homozygous canonical splice-site variant c.2064-1G>A in *DLGAP5*, supported by high sequencing depth (166×), affecting the −1 position of the intron 16 splice acceptor (Figure 1B). This variant is not observed in any individuals in gnomAD (v4.1) control population database. The aggregated score and results from *in silico* prediction tools, Splice AI (Δ score = 1), and Pangolin (Δ score = 0.89), is consistent in predicting that the variant may lead to potential alteration of splicing, thereby leading to transcript truncation with an overall loss-of-function effect. However, the precise transcript-level consequence could not be determined in the absence of RNA-based functional studies. A limitation of this study is the absence of experimental RNA-based validation of the *DLGAP5:* c.2064-1G>A splice site variant. No other pathogenic/likely pathogenic variant was observed in the couple WES (carrier screening test).

Validation and segregation analysis in the proband (II.2), parents (I.1 and I.2) and brother (II.3), of the family confirmed the homozygous state of the variant in the proband, heterozygous carrier status in father and mother, whereas the variant was not observed in the brother (Figure 1A). In the context of the patient’s highly specific reproductive phenotype and segregation in the family, the variant was classified as likely pathogenic (PVS1, PM2, PP4) under ACMG/AMP guidelines.

## Discussion

We report a woman with recurrent IVF failure characterized by poor oocyte maturation, reduced metaphase II (MII) oocyte yield, impaired embryo cleavage, and complete blastocyst arrest, in whom whole-exome sequencing identified a novel canonical splice-acceptor variant (c.2064-1G>A) in homozygous state in *DLGAP5*. Segregation analysis confirmed heterozygous carrier status in both parents, consistent with autosomal recessive inheritance. This case report provides first and additional evidence from Southern India of *DLGAP5*-related female infertility and expands the mutational spectrum of this recently recognized infertility gene.

Although recurrent ART failure is often attributed to maternal age or embryo aneuploidy, a growing number of cases have been traced to monogenic defects triggering female infertility. Reported genes include governing oocyte maturation (*PATL2*, *PANX1*), meiotic regulation and fertilisation (*WEE2*, *TLE6*, *BUB1B*, *STAG3*), zona pellucida structure (*ZP1*, *ZP2*, *ZP3*) and spindle and microtubule dynamics during meiosis and early cleavage (*TUBB8*, *CDC20*, *TRIP13*) (Solovova and Chernykh, 2022; Mordovina et al., 2024).

*DLGAP5* has recently emerged as a key regulator of human oocyte meiosis and early embryonic development (Hu et al., 2025; Qu et al., 2025; Wang et al., 2025). It encodes a Ran-GTP-dependent spindle-associated microtubule protein that is essential for bipolar spindle assembly, kinetochore microtubule stabilization, and faithful chromosome segregation during meiosis (Qu et al., 2025; Wang et al., 2025). Functional studies have shown that DLGAP5 localizes to the meiotic spindle in human oocytes, where its depletion results in disorganized spindle architecture, chromosome misalignment, reduced germinal vesicle breakdown, impaired polar-body extrusion, and defective oocyte maturation (Qu et al., 2025; Wang et al., 2025). More recently, DLGAP5 has been shown to interact with the spindle assembly factor TACC3 and to regulate oocyte maturation through the PI3K-AKT signalling pathway, providing mechanistic evidence linking *DLGAP5* deficiency to impaired meiotic progression and early embryonic development (Hu et al., 2025; Wang et al., 2026).

The clinical phenotype observed in our patient closely resembles the expanding spectrum (Figure 1C) of *DLGAP5*-associated infertility (Table 1). Qu et al. identified biallelic *DLGAP5* variants in infertile women with oocyte maturation arrest and demonstrated defective spindle assembly, reduced oocyte maturation, and impaired blastocyst development using human oocytes and knock-in mouse models (Qu et al., 2025). Consistent with these observations, our patient exhibited reproducible poor MII yield, impaired embryo cleavage, and complete blastocyst arrest across two independent IVF cycles, strongly supporting an intrinsic oocyte defect attributable to DLGAP5 dysfunction.

**TABLE 1:** Reported *DLGAP5*-associated female infertility cases and comparison with the present study.

Study	Family/patient	Age (years)	Consanguinity	*DLGAP5* variant(s)	Zygosity	ART cycles/method	Oocytes retrieved	MII/PB1 oocytes	Fertilization	Key embryological outcome
Qu et al., 2025	Family 1, patient II-1	28	Yes	c.431delA (p.Lys144Argfs*55)	Homozygous	IVF; IVF + ICSI; ICSI ×2	39 total	10/39 (25.6%)	8 fertilized overall	Predominantly OMA phenotype; only Grade III Day-3 embryos; no pregnancy after transfer
	Family 1, patient II-2	32	Yes	c.431delA (p.Lys144Argfs*55)	Homozygous	IVF ×2	6	3/6 (50.0%)	3 fertilized, with abnormal 3 PN fertilization reported	No usable embryos available
	Family 2, patient II-1	38	No	c.847C>G (p.Pro283Ala) + c.1202C>G (p.Thr401Ser)	Compound heterozygous	IVF ×2	9	9/9 (100%)	6/9 (66.7%)	Six usable Day-3 embryos; one good-quality blastocyst formed on day 5 but no pregnancy
Wang et al., 2025	Family 1, patient 1	NR	Yes	c.1101C>G (p.Tyr367*)	Homozygous	Multiple IVF and/or ICSI cycles	NR	Majority immature	NR	Slow cleavage with developmental arrest around day 3
	Family 1, patient 2 (sister)	NR	Yes	c.1101C>G (p.Tyr367*)	Homozygous	Multiple IVF and/or ICSI cycles	NR	Majority immature	NR	Recurrent ART failure with abnormal oocyte maturation
	Family 2, patient 1	NR	No	c.1101C>G (p.Tyr367*)	Homozygous	Three fresh IVF cycles	NR	Majority immature	No embryos available	Cycles terminated because of predominant oocyte immaturity
Hu et al., 2025	Family 1, patient 1	31	No	c.1101C>T (p.Tyr367Ter) and c.2469_2496del (p.Glu824Valfs*3)	Compound heterozygous	ICSI	24 across three attempts‡	11/16 evaluable (68.8%); first cycle MII NR	9/11 (81.8%) in evaluable cycles	Cleavage to day 3 occurred, but only two aneuploid blastocysts formed on day 5
	Family 2, patient 2	30	No	c.1101C>T (p.Tyr367Ter) and c.517C>T (p.Arg173Ter)	Compound heterozygous	IVF ×2	28	11/28 (39.3%)	7/11 (63.6%)	All embryos arrested at the 2–8-cell stage; no blastocysts
	Family 3, patient 3	31	Yes	c.1001A>G (p.Asn334Ser)	Homozygous	IVF + ICSI	13	7/13 (53.8%)	5/7 (71.4%)	One blastocyst formed on day 7; no pregnancy after transfer
Present study	Proband (II.2)	27	Yes	NM_014750.5:c.2064-1G>A	Homozygous	ICSI, two stimulated cycles	11 total	6/11 (54.5%)	5/6 (83.3%)	Abnormal oocyte morphology, poor cleavage, and complete failure of blastocyst formation

A limitation of this study is the absence of functional validation of the identified splice-site variant. RNA-based transcript analysis or a minigene splicing assay would provide direct evidence of aberrant splicing and further strengthen variant-specific pathogenicity. Nevertheless, the canonical splice-site location of the variant, segregation within the family, and the close phenotypic concordance with previously reported *DLGAP5*-associated infertility strongly support its pathogenic role (Hu et al., 2025; Qu et al., 2025; Wang et al., 2025; Wang et al., 2026).

Clinically, this case highlights the importance of early genomic evaluation in women with recurrent IVF failure characterized by low MII yield and consistent pre-blastocyst developmental arrest. Establishing a molecular diagnosis can prevent repeated unsuccessful and emotionally burdensome IVF cycles, facilitate accurate genetic counselling, and support informed reproductive decision-making. As additional cases are reported, further delineation of genotype–phenotype correlations and disease mechanisms will improve our understanding of *DLGAP5*-associated infertility and its implications for precision reproductive medicine.

## Data Availability

The original contributions presented in the study are publicly available. This data can be found in the ClinVar repository with the accession numbers VCV004884394.1.
