# Supplementary material for: A novel homozygous DLGAP5 splice-site variant in a woman with oocyte maturation arrest and recurrent IVF failure: a case report
Source: Front Genet. 2026 Sep 24;17:1963011. doi: 10.3389/fgene.2026.1963011 (PMC13639719; doi:10.3389/fgene.2026.1963011)
Supplement: Supplementary file 1 [file Table1.docx]

**Supplementary Table 1.** Summary of Assisted Reproductive Technology (ART) Outcomes in proband (II.2)

| **Parameter** | **Cycle 1** | **Cycle 2** |
| --- | --- | --- |
| Stimulation protocol | Antagonist protocol | Antagonist protocol |
| Duration of stimulation | 8 days | 9 days |
| Gonadotropin | Humog HP | HP-hMG (Menopur HP) |
| Daily gonadotropin dose | 300 IU/day | 300 IU/day |
| Antagonist | Cetrolix 0.25 mg/day for 4 days | Cetrolix 0.25 mg/day for 4 days |
| Estradiol on trigger day | 1206 pg/mL | 1214 pg/mL |
| Trigger agents | Ovitrelle 250 µg + Decapeptyl 0.1 mg | Ovitrelle 250 µg + Decapeptyl 0.1 mg |
| OPU timing after trigger | 35 hours | 35 hours |
| Fertilization method | ICSI | ICSI |
| Sperm sample | Fresh semen sample 1 from husband | Fresh semen sample 2 from husband |
| Sperm concentration on OPU day | 63 million/mL | 53 million/mL |
| Progressive motility on OPU day | 58% | 61% |
| Normal morphology | 3% | 3% |
| Oocytes retrieved | 6 | 5 |
| MII oocytes | 3 (50%) | 3 (60%) |
| Abnormal oocyte morphology | One oocyte with large polar body, enlarged perivitelline space, and cytoplasmic granularity | One oocyte with large/abnormal polar body and/or granular cytoplasm |
| Fertilized oocytes | 3/3 MII (100%) | 2/3 MII (66.7%) |
| Day 3 embryo development* | 7-cell Grade B; 5-cell Grade B; 2-cell Grade C | 2-cell Grade C; 2-cell Grade C |
| Blastocyst culture | Until Day 6 | Until Day 6 |
| Blastocyst formation | 0 | 0 |
| Overall outcome | No blastocyst formation | No blastocyst formation |

*Embryo morphology was assessed according to the ESHRE/ALPHA Istanbul Consensus criteria
